# Factors associated with the intention of telehealth service utilization among Bangladeshi people: a cross-sectional study

**DOI:** 10.12688/f1000research.124410.1

**Published:** 2022-09-05

**Authors:** Humayun Kabir, Md. Kamrul Hasan, Nahida Akter, U Swai Ching Marma, Tohidul Alam, Ariful Haque Tutul, Lila Biswas, Rawshan Ara, Dipak Kumar Mitra

**Affiliations:** 1 Department of Public Health, North South University, Dhaka, 1229, Bangladesh; 2 Department of Biochemistry and Molecular Biology, Tejgoan College, Dhaka, 1215, Bangladesh; 3 Penn State Ross and Carol Nese College of Nursing, Penn State University, University Park, Pennsylvania, PA 16802, USA; 4 International Organization for Migration, Cox's Bazar, Bangladesh; 5 Dhaka Medical College and Hospital, Dhaka, 1000, Bangladesh; 6 CRP Nursing College, Savar, 1343, Bangladesh; 7 Prime College of Nursing, Dhaka, 1229, Bangladesh

**Keywords:** Telehealth, telemedicine, knowledge, benefit, concern, predisposition, Bangladesh

## Abstract

**Background:** Telehealth is comprised of telecommunications and electronic information systems to support and maintain long-distance healthcare services. Although it has not been thoroughly explored, the intention of using the service among the general public is critical to its success. We investigated the factors associated with the intention to utilize telehealth services among the general population of Bangladesh.

**Methods:** This cross-sectional study was conducted between May 22, 2021 and June 15, 2021 in Bangladesh, where the total number of participants was 1038. The Pearson chi-square test and Kruskal-Wallis H tests were used to examine the unadjusted relationship between the explanatory variables and the intention to use telehealth services. A multinomial logistic regression model was fitted to determine the adjusted association. Shapiro-Wilk tests were used to check the normality of continuous data. Data were processed and analyzed by software STATA-16.

**Results:** The probability of utilizing the service increased significantly with increasing knowledge, perceived benefit, and predisposition levels among respondents. However, when perceived concern increased, the likelihood of utilizing the service dropped significantly. Age, marital status, educational status, profession, residence, and perceived health status were significantly associated with the participants’ intention to utilize the telehealth service.

**Conclusions:** The influencing aspects of telehealth service utilization should be recognized by the respective authorities. Possible activities to enhance usability among people are also recommended.

## Introduction

Over the last few decades, Bangladesh’s healthcare system has made significant advancements; in particular, made tremendous progress in public health while focused on meeting the Millennium Development Goal. ^1^ It has also had considerable success in lowering maternal and newborn mortality and morbidity rates, as well as diarrheal illnesses, malaria, and tuberculosis. ^2^ However, challenges still exist, such as Bangladesh is staying a long way from obtaining universal health care for all from vulnerable groups, e.g., low socio-economic status and people from remote locations. ^3^ The shortages of skilled healthcare personnel, climate-induced environmental changes, ^4^ and transportation constraints are the most pressing issues in accessing the healthcare system. ^5^ Telehealth, in which people have benefited from recent technological advancements, has the potential to overcome many of these health care system-related constraints. ^3^

Telehealth uses telecommunications and electronic information to support and continue long-distance clinical health service, healthcare management, and patient-focused health education. ^6^ This term also refers to a wide range of remote healthcare service delivery strategies. Telehealth is recommended as one of the most essential and promising technology-based services for addressing many challenges the Bangladeshi healthcare system usually faces. ^7^ Nevertheless, the effectiveness of the telehealth service is contingent on healthcare professionals’ and the general public’s acceptance and willingness to utilize it. ^8^ As individuals can be benefited from how telehealth services are offered, ^9^ it is crucial to understand their intentions and the significance of other elements in their decision to utilize telehealth to build services that people will embrace. ^9^

However, the intention of the general population to utilize telehealth services was not widely documented; the literature generally focuses on physicians’ and clients’ impressions of telehealth services. There were a few reports on peoples’ attitudes, but they did not focus on the elements that can influence their decision to adopt telehealth. Jennett et al. (2003) conducted a study in Canada to assess the preparation of rural people to utilize telehealth, ^10^ and Schwarz et al. (2014) conducted a study in New South Wales, Australia, to investigate the preparedness of Australia’s distant population. ^11^ However, both studies drew their samples from key informants or persons who had already used telehealth services rather than the general population. Likewise, several studies conducted in different places in the world emphasized the physician’s views toward telehealth, but general people’s perceptions or intentions toward telehealth service utilization were merely investigated. ^12^^–^^14^

In Bangladesh, the majority of telehealth research has focused on the role and importance of telehealth service, ^15^^,^^16^ how it may be used in disease or outbreak management, ^17^ and methods to promote the telehealth sector. ^18^ Furthermore, few pieces of research focused on healthcare providers’ perceptions of telemedicine and potential hurdles in Bangladesh. ^19^^,^^20^ Those studies only included specific samples yet focused on the general people’s intentions. Despite the fact that a study described the people’s economic view ^7^ and the perception of people with chronic diseases, they have yet to examine the influence of demographic or health-related factors on intention to utilize the telehealth service. ^7^^,^^21^ Therefore, this research investigated the factors associated with the intention to utilize telehealth services among the general population of Bangladesh.

## Methods

### Study design, participants, and setting

This cross-sectional study was conducted during the coronavirus disease 2019 (COVID-19) pandemic between May 22, 2021, and June 15, 2021. In this study, 1038 Bangladeshi population participated. The inclusion criteria for the study participants was at least 18 years old and willing to participate by ensuring consent. Those who failed to provide the completed responses to the questionnaire were excluded from this study.

### Study variables

The outcome variable of the study was the intention of utilizing telehealth services. The respondents’ were asked to respond to an item; “ *Do you have any plan to utilize telehealth service in the future?*” The responses were considered into three categories such as “they will not utilize,” “they may utilize” and “they will utilize”. The exploratory variables included participants’ knowledge, perceived benefit, perceived concern, and predisposition to telehealth, demographic variables (age, sex, marital status, educational status, profession, residence, and division), and perceived health status.

### Knowledge, perceived benefit, perceived concern, and predisposition

The knowledge, perceived benefit, perceived concern, and predisposing were measured by adopting a questionnaire by Gagnon et al. (2004), who conducted a study in Quebec, Canada. ^8^ The questionnaire was developed based on the telehealth applications such as telediagnosis, telemonitoring, teletriage, teleintervention, remote access, continuing education for health care professionals, cost savings, quality, safety, and confidentiality. The item of telehealth knowledge was a response to a three-point scale. All the responses to the items related to perceived benefit, perceived concern, and predisposition was obtained via a five-point scale. The details of the questionnaire are provided in the extended data. ^49^

### Perceived health status

To evaluate perceived health status, we implemented a single-item measure suggested by the World Health Organization (WHO) ("In general, how would you rate your current health status?"). ^22^^,^^23^ The scale featured five options for replies; “very good”, “good”, “fair”, “bad”, or “very bad”. For statistical purposes, “very good” and “good” were considered “good”, while “fair” was regarded as “as usual”. ^23^ Similarly, “poor” was regarded to be “bad” and “very bad”. A similar scale was used to measure the Bangladeshi healthcare workers’ perceived health status. ^24^

### Questionnaire development and data collection

For this study, a structured questionnaire was developed. Two experts reviewed the questionnaire, and required changes were performed based on their recommendations. The completed questionnaire was transferred to “Google Form” in terms of creating an online version. By utilizing an online questionnaire, data were acquired, and a convenient sampling procedure was followed. The questionnaire was distributed via social media platform (Facebook and WhatsApp) with the request that respondents participate voluntarily. By using these strategies, after receiving 1130 responses, 1038 completed responses were accepted for data analysis.

### Sample size

Our estimated sample size was 784 at 80% power, 95 % CI of 0.05 to 1.96, and 3.5% margin of error, with the assumption that 50% of study participants were knowledgeable about telehealth. ^25^ The authors aimed to reduce the margin of error by collecting a larger sample size than was required, and an additional 254 responses (32% of the estimated sample size) were included. As a result, a total of 1038 samples were included in the final analysis.

### Statistical analysis

Data were automatically entered into an online Excel spreadsheet considering the nature of data collection. Only completed responses were collected and processed into the data analysis software STATA-16. The Pearson chi-square test was used to examine the unadjusted relationship of demographic and perceived health status with the intention to utilize telehealth services. Furthermore, Shapiro-Wilk tests were used to check the normality of knowledge, perceived benefit, perceived concern, and predisposition score and found that the scores were non-normally distributed. Kruskal-Wallis H test is usually used to assess more than two independent subgroups with non-normally distributed data. As a result, the Kruskal-Wallis H test was used to determine the unadjusted association between the scores and the intention to utilize telehealth services. Finally, a multinomial logistic regression model was fitted to determine the adjusted association between the explanatory variables and the intention to utilize telehealth services. The *p*-value of 0.05 was considered statistically significant at the 95% confidence interval.

### Ethical considerations

On the top of the page of the questionnaires, the study aims and objectives were specifically outlined. The authors secured the privacy of the acquired data and the respondents’ flexibility to exit from the study at any moment. This study’s subjects were all Bangladeshi, and none were under the age of 18 years. Besides, the respondents were asked for electronic signatures; after that, they were asked to respond and submit the answers to the items. Moreover, the Ethical Review Committee of Tejgoan College, Dhaka-1215, Bangladesh, reviewed and approved the study on April 25, 2021 (reference number 2021/OR-TGC/0202). The research was carried out in line with the Helsinki Declaration. ^26^

## Results

### Sample

**Figure 1.**
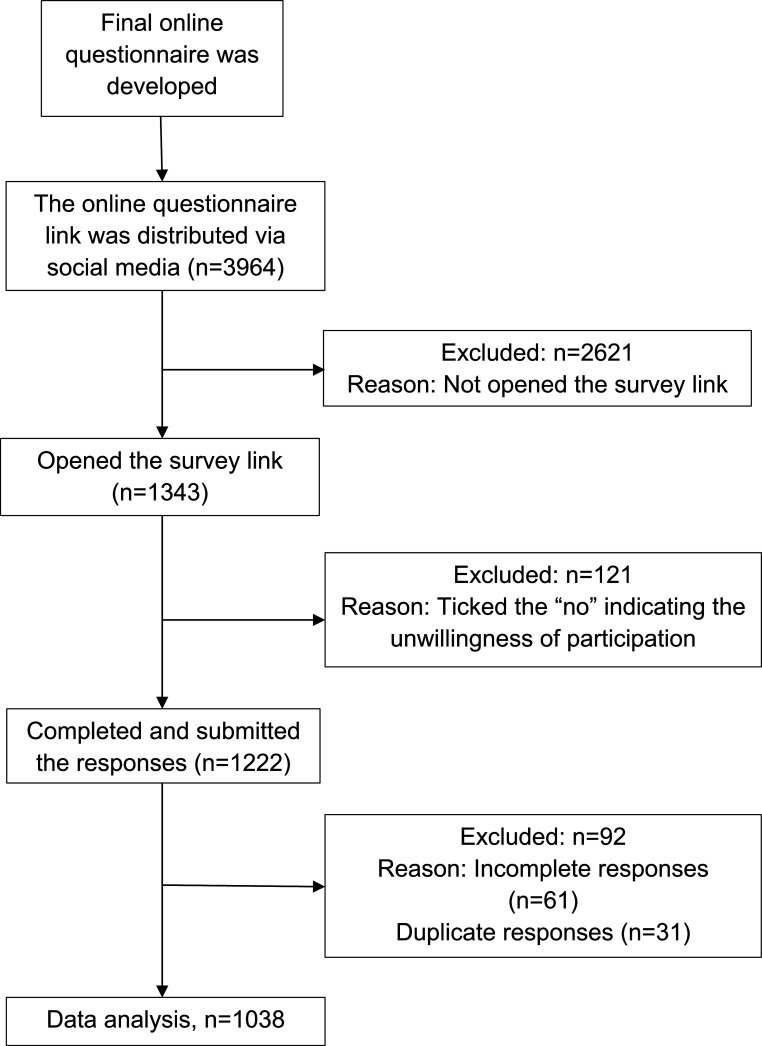
Flow diagram of sample recruitment procedure.

A flow diagram of the sample recruitment procedure in detail is presented in Figure 1. In total, 1038 responses were analysed.

### Distributions of demographic and perceived health status by intention to utilize telehealth service

In Table 1, the distributions of demographic and perceived health status by intention to utilize telehealth services are presented. ^48^ In the age group of 30-39 years, 40.83% intended to utilize (will utilize) the telehealth service. The number of unmarried participants intended to utilize the telehealth service was 37.72%, whereas the intention of utilizing the service among graduates was 44.00%. The urban residence’ intention to utilize the service was 38.57%. Of those who perceived poor health status, their intention to utilize the service was 38.35%.

**Table 1.** Distributions of demographic and perceived health status by intention to utilize telehealth service.

Variables	Intention to utilize telehealth service			χ ^2^	*p*-value
	They will not utilize	They may utilize	They will utilize		
**Demographic variables**					
**Age**					
<20 years	9 (42.68)	38 (46.34)	35 (42.68)	26.07	**<0.001**
20-29 years	61 (9.40)	339 (52.23)	249 (38.37)		
30-39 years	20 (16.67)	51 (42.50)	49 (40.83)		
≥40 years	38 (20.32)	100 (53.87)	49 (26.20)		
**Sex**					
Male	70 (12.41)	286 (50.71)	208 (36.88)	0.01	0.993
Female	58 (12.24)	242 (51.05)	174 (36.71)		
**Marital status**					
Married	64 (16.04)	194 (48.62)	141 (35.34)	8.25	**0.016**
Unmarried	64 (16.02)	334 (52.27)	241 (37.72)		
**Educational status**					
Graduate	29 (7.25)	195 (48.75)	176 (44.00)	55.00	**<0.001**
HSC	50 (10.99)	256 (56.26)	149 (32.75)		
Up to SSC	49 (26.78)	77 (42.08)	57 (31.15)		
**Profession**					
Student	47 (9.07)	273 (52.70)	198 (38.22)	10.15	**0.006**
Employed	81 (15.58)	255 (49.04)	184 (35.38)		
**Residence**					
Rural	27 (18.49)	81 (55.48)	38 (26.03)	11.30	**0.004**
Urban	101 (11.32)	447 (50.11)	344 (38.57)		
**Division**					
Dhaka	103 (12.34)	420 (50.30)	312 (37.37)	0.64	0.726
Other than Dhaka	25 (12.32)	108 (53.20)	70 (34.48)		
**Perceived health status**					
Poor	82 (10.73)	389 (50.92)	293 (38.35)	9.12	0.058
As usual	30 (15.63)	101 (52.60)	61 (31.77)		
Good	16 (19.51)	38 (46.34)	28 (34.15)		

SSC = Secondary school certificate, HSC = Higher secondary school certificate.

The bold *p*-value indicates the statistically significant variables.

### Distributions of knowledge, perceived benefit, perceived concern, and predisposition by intention to utilize telehealth service

In Table 2, the distributions of knowledge, perceived benefit, perceived concern, and predisposition by intention to utilize telehealth services are presented. The knowledge, perceived benefit, and predisposition scores were significantly highest (p<0.001) among those who will utilize the telehealth service. Albeit, the perceived concern score was found to be significantly highest (p<0.001) among those who will not utilize the telehealth service.

**Table 2.** Distributions of knowledge, perceived benefit, perceived concern, and predisposition by intention to utilize telehealth service.

Variables	Intention to utilize telehealth service						*p*-value
	They will not utilize		They may utilize		They will utilize		
	Mean (SD)	IQR	Mean (SD)	IQR	Mean (SD)	IQR	
**Knowledge**	11 (3.09)	5-15	12.84 (2.53)	5-15	14.10 (1.84)	14-15	**<0.001**
**Perceived benefit**	13.72 (3.56)	12-16	15.92 (2.95)	7-20	17.81 (2.34)	11-20	**<0.001**
**Perceived concern**	3.45 (1.27)	3-5	2.84 (1.15)	2-4	2.5 (1.27)	1-3	**<0.001**
**Predisposition**	6.23 (2.21)	5-8	7.81 (1.73)	6-9	8.91 (1.41)	8-10	**<0.001**

The bold *p*-value indicates the statistically significant variables.

### Factors associated with the intention to utilize telehealth service

Table 3 presented a multinomial logistic regression that was used to determine the factors (sociodemographic factors and perceived health status) associated with the intention of utilizing telehealth services. The reference category of the outcome variable was “they will not utilize,” and each of the other two (“they may utilize” and “they will utilize”) was compared to this reference group. The probability of utilizing the service increased significantly with increasing knowledge level among respondents who ‘may utilize’ (RRR= 1.24, 95% CI: 1.16-1.32) and ‘will utilize’ (RRR= 1.64, 95% CI: 1.50-1.80). Similarly, as the perceived benefit score climbed significantly, so did the likelihood of utilizing the service among those who ‘may utilize’ (RRR= 1.22, 95% CI: 1.15-1.30) and those who ‘will utilize’ (RRR= 1.62, 95% CI: 1.50-1.75). However, when perceived concern increased, the likelihood of utilizing the service dropped significantly among respondents who ‘may utilize’ (RRR= 0.67, 95% CI: 0.57-0.79) and ‘will utilize’ (RRR= 0.53, 95% CI: 0.45-0.63). Albeit, the chance of utilizing service increased with the increased level of predisposition among respondents who ‘may utilize’ (RRR= 1.47, 95% CI: 1.33-1.63) and ‘will utilize’ (RRR=2.30, 95% CI: 2.02-2.61). In comparison to the oldest age group (≥40 years), the chance of utilizing the service was 3.02 (RRR=3.02, 95% CI: 1.29-7.03) times significantly higher among the youngest (<20 years). Furthermore, the likelihood of ‘may utilize’ and ‘will utilize’ the service were 2.11 (RRR=2.11, 95% CI: 1.13-3.35) times and 3.17 (RRR=3.17, 95% CI: 1.91-5.26) times among the participants with age of 20-29 years. Compared to the married, the unmarried participants were significantly 72% (RRR=1.72, 95% CI: 1.17-2.54) and 71% (RRR=1.71, 95% CI: 1.14-2.56) more likely to ‘may utilize’ and ‘will utilize’ the service, respectively. The graduate participants were 4.28 (RRR=4.28, 95% CI: 2.52-7.27) times and 5.22 (RRR=5.22, 95% CI: 3.02-9.02) times more likely to ‘may utilize’ and ‘will utilize’ the service, compared to the education level up to SSC. Similarly, HSC completed respondents’ intention to utilize service in the category of ‘may utilize’ and ‘will utilize’ was 3.26 (RRR=3.26, 95% CI: 2.04-5.51) times and 2.56 (RRR=2.56, 95% CI: 1.56-4.22) times higher than the up to SSC level educated. Among the students’ the possibility of ‘may utilize’ and ‘will utilize’ the service was 85% (RRR=1.85, 95% CI: 1.24-2.75) and 86% (RRR=1.86, 95% CI: 1.23-2.80) higher compared to the employed respondents. The urban residence was 2.42 (RRR=2.42, 95% CI: 1.41-4.16) times more likely to ‘will utilize’ service than the rural.

**Table 3.** Multinomial logistic regression analysis to find the factors associated with the intention to utilize telehealth service.

Variables	Intention to utilize telehealth service					
	They may utilize			They will utilize		
	RRR	95% CI	*p*-value	RRR	95% CI	*p*-value
**Knowledge**	1.24	1.16-1.32	**<0.001**	1.64	1.50-1.80	**<0.001**
**Perceived benefit**	1.22	1.15-1.30	**<0.001**	1.62	1.50-175	**<0.001**
**Perceived concern**	0.67	0.57-0.79	**<0.001**	0.53	0.45-0.63	**<0.001**
**Predisposition**	1.47	1.33-1.63	**<0.001**	2.30	2.02-2.61	**<0.001**
**Age**						
<20 years	1.60	0.71-3.63	0.257	3.02	1.29-7.03	**0.011**
20-29 years	2.11	1.13-3.35	**0.002**	3.17	1.91-5.26	**<0.001**
30-39 years	0.97	0.51-1.82	0.923	1.90	0.97-3.72	0.061
≥40 years	Reference			Reference		
**Sex**						
Male	Reference			Reference		
Female	1.02	0.69-1.51	0.915	1.01	0.68-1.51	0.963
**Marital status**						
Married	Reference			Reference		
Unmarried	1.72	1.17-2.54	**0.006**	1.71	1.14-2.56	**0.009**
**Educational status**						
Graduate	4.28	2.52-7.27	**<0.001**	5.22	3.02-9.02	**<0.001**
HSC	3.26	2.04-5.51	**<0.001**	2.56	1.56-4.22	**<0.001**
Up to SSC	Reference			Reference		
**Profession**						
Student	1.85	1.24-2.75	**0.003**	1.86	1.23-2.80	**0.003**
Employed	Reference			Reference		
**Residence**						
Rural	Reference			Reference		
Urban	1.48	0.91-2.40	0.117	2.42	1.41-4.16	**0.001**
**Division**						
Dhaka	Reference			Reference		
Other than Dhaka	1.06	0.65-1.72	0.816	0.92	0.56-1.54	0.762
**Perceived health status**						
Poor	2.00	1.06-3.75	**0.032**	2.04	1.05-3.96	**0.034**
As usual	1.42	0.70-2.89	0.337	1.16	0.55-2.47	0.696
Good	Reference			Reference		

RRR = Relative risk ratio, SSC = Secondary school certificate, HSC = Higher secondary school certificate.

The bold *p*-value indicates the statistically significant variables.

## Discussion

Several pieces of research around the world have already shown how people’s and health care providers’ views and intentions influence their usage of telehealth services. ^12^^–^^14^^,^^19^^,^^20^ Meanwhile, few pieces of research in Canada looked at the impact of social, cultural, economic, and environmental factors on people’s decisions to utilize telehealth-care services. ^27^^,^^28^ In Bangladesh, the intention of telehealth service utilization of the general population was yet to be investigated. Therefore, in this study, we examined the associated factors such as sociodemographic factors, perceived health status, knowledge, perceived benefit, perceived concern, and predisposition with the intention to utilize telehealth services.

Our study found a significant association between telehealth of knowledge and the intention to utilize the service. We found that as the knowledge score improved, the level of intention to utilize the telehealth service increased. Albarrak et al. (2021) reported that Saudi Arabian participants had an average level of understanding were more intended to utilize the telehealth service. ^29^ Albarrak et al. (2021) believed that training on telehealth could improve understanding, and this was expected to influence people’s intention to utilize the telehealth service. ^29^ Malhotra et al. (2020) in India and Woo & Dowding (2019) in the United States also drew a similar conclusion as our study findings. ^30^^,^^31^

In our study, participants were more intent to utilize the telehealth services if they perceived a high level of benefit. Similarly, Alaboudi et al. (2016) and El-Mahalli et al. (2012) in Saudi Arabia found the same result that the participants who perceived the benefit of the service were more intended to utilize the service. ^32^^,^^33^ When participants had a high level of perceived concern, their intention to utilize this service was found low. Numerous studies around the world found that worried and particularly privacy issues were connected to the intention of technology-based health service utilization. ^34^^,^^35^ Furthermore, Bangladeshis who had a favorable predisposition were more interested in the telehealth service. In order to determine whether socio-demographics and technology-enabling factors may influence how telehealth is utilized, a study was carried out in the United States in 2021. ^36^ The researchers revealed that individuals with favorable predispositions toward ICT devices and internet access exhibited more enthusiasm for using telehealth. ^36^

We found that several demographic factors were significantly associated with the intention to utilize telehealth services. Age influenced people’s intention to adopt telehealth services. The intention to utilize the service in the near future was 3.02 times higher among the youngest (<20 years) than it was among the oldest (≥40 years). Numerous studies also supported the findings of our research. Lee et al. (2019) and Raghunathan et al. (2018) found that younger participants had a stronger intention to utilize telemedicine or internet adaptive equipment, which could be due to their competence and past familiarity with the advanced technology. ^37^^,^^38^ However, Jenkins et al. (2016) reported that older stroke patients were more eager to employ telehealth observation with ongoing technical assistance than younger patients were. ^39^

Our research focused on education status and found that those with better education were more likely to be utilizing telehealth services. More specifically, we found that graduate participants were 4.28 and 5.22 times more likely to “may utilize” and “will utilize” the service, respectively, than those with an education level up to SSC. Schulz et al. (2013) found that persons with higher levels of education were more likely to pay for and use technology-based health monitoring systems. ^40^ Another study found that cancer patients with higher education were more likely to use mobile devices to utilize their health care services. ^38^ Education may frequently be mentioned as a component that influences people’s thoughts and actions, but few researchers have looked at this factor in the interaction between the fundamental indicators and people’s willingness to adopt technology-based health monitoring systems. ^41^

Our study revealed that unmarried participants were much more intent to utilize telehealth than married people were; 72% and 71% ‘may utilize’ and ‘will utilize’ the service, respectively. According to Choi et al. (2021) telehealth users were more likely to be married or in a relationship than non-users. ^36^ Additionally, they demonstrated in their research that during the COVID-19 outbreak, a more significant percentage of users than non-users either joined in with others or somebody came in with them. ^36^

The intention was also found higher among students than among other employed individuals. The possible reason could be as they were more likely to expose to distance learning and technology based service usability. ^42^^–^^45^ A similar study measuring nursing students’ readiness to use telenursing was carried out in Poland. ^46^ Amazingly, 69.49% of the students reported that they would be very likely to include telenursing in their course curriculum. ^46^ Additionally, students from a selected number of colleges demonstrated a noticeably greater desire to integrate telenursing courses into the nursing curriculum and a willingness to utilize telenursing services in their future professional practice. ^46^

Furthermore, we found that urban people were more intent on utilizing this service compared to rural individuals. However, according to Lin et al. (2004) the people of rural areas had a higher likelihood of adopting telehealth services. ^47^ Moreover, in Canada, Gagnon et al. (2004) did not find any influence of residential status on participants’ intention of telehealth service utilization. ^8^

An association between perceived health status and the intention to utilize the telehealth service was found in our study. Our findings revealed that those with a low health status were more intended to utilize telehealth services than those with normal or good health status. Similarly, Ghaddar et al., (2020) conducted a study in the United States and found that participants with chronic diseases were more interested in utilizing the telehealth service than those with good perceived health status. ^3^

## Conclusions and recommendations

The healthcare system seems successful when there is equal participation in services. However, there are many constraints that may hinder people from getting access to the universal health system. Therefore, several initiatives can be considered to reach the health service to the people’s door, including the technology-based digital health system telehealth. Without addressing the digital constrain of accessing the existing healthcare system, the disparities at the community level may be unprivileged. Numerous factors were identified to be connected with people’s intention to utilize the telehealth service in this study. The appropriate authorities can consider the factors and conduct studies more broadly to investigate more containment of telehealth implementation. Moreover, we advocated for policy actions to make the telehealth system more accessible to the community and to enhance the understanding and awareness of the benefits of utilizing digital health to improve usability among the underprivileged.

### Strengths and limitations

Telehealth is an emerging healthcare platform that allows patients to access healthcare services effortlessly. Public intent to utilize the platform is also deemed significant when encountering an expansion of this service widely. However, no study had yet investigated the intention of telehealth among Bangladeshi adults, which is one of the strengths of this study. The large sample size in this study provides substantial support for the accuracy of the results. However, selection bias was inescapable due to the convenient nature of data collection method. Because of the cross-sectional study design, causality between variables could not be established. Moreover, more rigorous research is recommended in Bangladesh by utilizing a random sampling approach.

## Data availability

### Underlying data

Zenodo: Intention to utilize telehealth service in Bangladesh, https://doi.org/10.5281/zenodo.6862986. ^48^

This project contains the following underlying data:

• Intention_utilize_telehealth_bd.xls (raw data). Data set of the study.

### Extended data

Zenodo: Telehealth research questionnaire in detail, https://doi.org/10.5281/zenodo.6953539. ^49^

This project contains the following extended data:

• Telehealth_research_questionnaire.docx (in English). Copy of the research questionnaire.

Data are available under the terms of the Creative Commons Attribution 4.0 International license (CC-BY 4.0).
