# Supplementary material for: Factors associated with the intention of telehealth service utilization among Bangladeshi people: a cross-sectional study
Source: F1000Res. 2024 Feb 27;11:996. Originally published 2022 Sep 5. [Version 2] doi: 10.12688/f1000research.124410.2 (PMC10940848; doi:10.12688/f1000research.124410.2)
Supplement: Peer review history

# Reviewer report: Reviewer response for version 2

**DOI:** 10.5256/f1000research.162850.r250657

**Published:** 2024-04-04

**Authors:** Victoria Ramos

**Affiliations:** 1 Telemedicine and Digital Health Research Unit, Instituto de Salud Carlos III, Madrid, Spain

I agree with new version. From my point of view, it is ready.

Is the work clearly and accurately presented and does it cite the current literature?

Yes

If applicable, is the statistical analysis and its interpretation appropriate?

Partly

Are all the source data underlying the results available to ensure full reproducibility?

Yes

Is the study design appropriate and is the work technically sound?

Yes

Are the conclusions drawn adequately supported by the results?

Partly

Are sufficient details of methods and analysis provided to allow replication by others?

Yes

Reviewer Expertise:

NA

I confirm that I have read this submission and believe that I have an appropriate level of expertise to confirm that it is of an acceptable scientific standard.

# Reviewer report: Reviewer response for version 2

**DOI:** 10.5256/f1000research.162850.r250656

**Published:** 2024-03-14

**Authors:** Michelle Lozada-Urbano

**Affiliations:** 1 South American Center for Education and Research in Public Health, Universidad Privada Norbert Wiener, Lima, Peru

The authors have met the suggestions for improvement

Is the work clearly and accurately presented and does it cite the current literature?

Yes

If applicable, is the statistical analysis and its interpretation appropriate?

I cannot comment. A qualified statistician is required.

Are all the source data underlying the results available to ensure full reproducibility?

Yes

Is the study design appropriate and is the work technically sound?

Yes

Are the conclusions drawn adequately supported by the results?

Partly

Are sufficient details of methods and analysis provided to allow replication by others?

Yes

Reviewer Expertise:

Nutrition, Public Health and Health economic

I confirm that I have read this submission and believe that I have an appropriate level of expertise to confirm that it is of an acceptable scientific standard.

# Reviewer report: Reviewer response for version 1

**DOI:** 10.5256/f1000research.136599.r222057

**Published:** 2024-01-22

**Authors:** Michelle Lozada-Urbano

**Affiliations:** 1 South American Center for Education and Research in Public Health, Universidad Privada Norbert Wiener, Lima, Peru

This study is important, as it can help understand how to improve access to care for patients who are not close to health services. It is necessary to modernize the health system. The use of telehealth has shown to be efficient in patient care, knowing the intention of use and the associated factors, which is the objective of this study, is key to understanding where the best application could be, together to other studies such as satisfaction and evaluation.                   

 When mentions the use of a questionnaire by Gagnon et al. It is better to place if you have internal and external validation.

 In table 1, the columns express the number (percentage), you can enter n(%).

 I would like to know that they only have 3 categories of educational level. In the case of graduates, it would have been important to know if they are from the health area or from other careers. This can help to better understand the behavior of these variables, as mentioned in the discussion. about nursing students.

 They mention cancer patients as being more predisposed to using virtual care; other studies during the COVID-19 pandemic have demonstrated optimal care for overweight and obese patients, with anthropometric and nutritional changes. Students today are familiar with these new technologies or telehealth. This comment may help improve the limitations.

 I think the conclusions and recommendations could be improved. There is an understudied population that is older adults who have movement restrictions and have caregivers in their care.

Is the work clearly and accurately presented and does it cite the current literature?

Yes

If applicable, is the statistical analysis and its interpretation appropriate?

I cannot comment. A qualified statistician is required.

Are all the source data underlying the results available to ensure full reproducibility?

Yes

Is the study design appropriate and is the work technically sound?

Yes

Are the conclusions drawn adequately supported by the results?

Partly

Are sufficient details of methods and analysis provided to allow replication by others?

Yes

Reviewer Expertise:

Nutrition, Public Health and Health economic

I confirm that I have read this submission and believe that I have an appropriate level of expertise to confirm that it is of an acceptable scientific standard, however I have significant reservations, as outlined above.

## References

[1] : The effects of telenutrition in overweight and obese adults in a nutritional center in Lima, Peru. *F1000Res* .2021;10: 10.12688/f1000research.53564.2 545 10.12688/f1000research.53564.2 34950451 PMC8666983

[2] : Case Management and Telehealth: A Scoping Review. *Telemed J E Health* .2022;28(1) : 10.1089/tmj.2021.0012 11-23 10.1089/tmj.2021.0012 33847524

# Reviewer report: Reviewer response for version 1

**DOI:** 10.5256/f1000research.136599.r149710

**Published:** 2022-10-21

**Authors:** Victoria Ramos

**Affiliations:** 1 Telemedicine and Digital Health Research Unit, Instituto de Salud Carlos III, Madrid, Spain

Good and innovative work in Bangladesh where the intention of telehealth among Bangladeshi adults had not been studied before and is important to improve usability among the underprivileged.

 Some contrariety in the expression. For instance, this work revealed that unmarried participants were much more intent on utilizing telehealth than married people were, but it does not take into account another work that presents that telehealth users were more likely to be married or in a relationship than non-users. Could there be a relationship between the intent to use telehealth services and the need for socialization or loneliness?

 Unexpected result about urban people being more intent on utilizing this service compared to rural individuals when it is taken into account that it is probable that, in urban environments, there is more access to healthcare service than in rural environments.

 A review of the discussion of the results is recommended. **Their findings revealed that those with a low health status had more intention to utilize telehealth services than those with normal or good health status, without any reason. On the contrary, the authors believe that severe cases prefer face-to-face care over telecare.

 There are a number of issues with the methods and analysis that are not clear enough. Below are some comments:

 I have indicated that there is some contradiction between the intention of the married and the unmarried. In the second paragraph on page 9, there seems to be a contradiction and it would be interesting if they give some explanation about it.

 It would also improve the work to have some indications on how to improve the intention to use among those less favorable to telecare. The young and digitally born are already immersed in these tools and have no problem using them. Is there any explanation for that perceived concern? When concern increases, the likelihood of utilizing the service dropped significantly.

Is the work clearly and accurately presented and does it cite the current literature?

Yes

If applicable, is the statistical analysis and its interpretation appropriate?

Partly

Are all the source data underlying the results available to ensure full reproducibility?

Yes

Is the study design appropriate and is the work technically sound?

Yes

Are the conclusions drawn adequately supported by the results?

Partly

Are sufficient details of methods and analysis provided to allow replication by others?

Yes

Reviewer Expertise:

Telemedicine and Digital health

I confirm that I have read this submission and believe that I have an appropriate level of expertise to confirm that it is of an acceptable scientific standard.
